# Amateur ballet practicing, body image and eating behaviors: a comparative study of classical ballet dancers, gym users and sedentary women

**DOI:** 10.1186/s40337-021-00459-9

**Published:** 2021-08-28

**Authors:** Ruty Eulália de Medeiros Eufrásio, Rannapaula Lawrynhuk Urbano Ferreira, Leilane Lílian Araújo Leal, Erikarla Baracho Avelino, Rafaela Nayara da Costa Pelonha, Maria Clara da Cruz Carvalho, Carlos Henrique de Medeiros Torres, Ana Luísa de Sousa Praseres, Marcos de Paiva Lima Filho, Ana Carolina Costa Campos Mota, Adriana Bezerra Nunes, Diana Quitéria Cabral Ferreira, Sancha Helena de Lima Vale, Bruna Leal Lima Maciel

**Affiliations:** 1 Nutrition Department, Federal University of Rio Grande Do Norte, Avenue Senador Salgado Filho 3000, Lagoa Nova, Natal, RN 59078-970 Brazil; 2 Postgraduate Nutrition Program, Federal University of Rio Grande Do Norte, Natal, RN Brazil; 3 Department of Clinical Medicine, Onofre Lopes University Hospital, Federal University of Rio Grande Do Norte, Natal, RN Brazil

**Keywords:** Body image dissatisfaction, Body image distortion, Anorexia nervosa, Bulimia nervosa, Dancers

## Abstract

**Background:**

Ballet dancers are a risk group for body image (BI) distortion, dissatisfaction and eating disorders (ED), but few studies have investigated these aspects in amateur adult practitioners. This study aimed to evaluate if amateur female adult classical ballet dancers presented different BI and behaviors for ED than gym users and sedentary women.

**Methods:**

This is a cross-sectional study where classical ballet dancers (n = 19) were compared to gym users (n = 19) and sedentary women (n = 19). Body mass index (BMI) was assessed, and a figure rating scale was applied to assess BI distortion/dissatisfaction. The body shape questionnaire (BSQ) was used to measure BI concern. The eating attitudes test (EAT-26) and the bulimic investigatory test, Edinburgh (BITE) were used for behaviors toward anorexia and bulimia.

**Results:**

BMI was significantly lower in ballet dancers than gym users and sedentary women (*F, p* = .04). BI distortion did not differ among the studied groups. BI dissatisfaction was lower (*X*^*2*^*, p* = .041) in ballet dancers (75.0%) and gym users (70.6%) compared to sedentary women (100%). Correspondence analysis showed ballet dancers were mostly not concerned with BI, which was not observed among the other groups. The EAT-26 did not differ between the studied groups. The BITE score was lower (Tukey’s post hoc test, *p* = .005) in the ballet dancers [mean 5.3 (5.6)] compared to the sedentary women [mean 10.9 (4.8)].

**Conclusions:**

Data suggest that amateur classical ballet practicing is associated to better BI and fewer behaviors for ED in the studied population. The lower BMI in ballet dancers might explain these findings, and further studies should explore these associations.

## Background

In ballet, a lean body with low body fat is considered aesthetically essential to perform the movements [1, 2]. These body characteristics may put ballet dancers under pressure to maintain the desired body shape and can possibly affect body image (BI) [3]. BI refers to thoughts and feelings about the size, contour and shape of the body [4]. Distortion, dissatisfaction, and concern with BI, and also eating behaviors are triggering factors for the development of eating disorders (ED) [5], a group of mental illnesses characterized by abnormal eating habits [6].

It is widely accepted that BI concern in ballet dancers is higher than in the general population [3, 7], especially in the professional environment, where there is pressure to maintain a standard body, and BI dissatisfaction tends to be higher [8]. Higher BI concern, dissatisfaction [8, 9], and distortion [10] is especially present in the professional ballet environment, where there is pressure to maintain a lean body, increasing the risk for ED [8, 9, 11–13].

Sports that emphasize low weight for exemplary performance in women, such as classical ballet, are associated with a higher prevalence of the female athlete triad. The triad includes a spectrum of dysfunctions involving imbalanced energy availability, menstrual dysfunctions, compromised bone mineral density (BMD), and are common in professional ballet dancers [14, 15]. Nonetheless, low BMD has been observed even in non-professional ballet dancers and is associated with eating disordered behaviors and BI [46–48], and studies should explore these relationships more.

A meta-analysis has estimated that the overall prevalence of ED in ballet dancers was 16.4%, being 2.0% for anorexia and 14.9% for bulimia [16]. In professional ballet dancers, the frequency of eating disordered behaviors is higher amongst those with BI dissatisfaction [3, 8, 17]. Studies have also found BI dissatisfaction in amateur classical ballet dancers [18, 19]. Although most of these ballet dancers did not reach the professional stage, they showed great concern with physical fitness, which could lead to BI distortion [19]. Thus, even amateur classical ballet dancers may suffer from the pressures for an aesthetic pattern suitable for dance, even if in a smaller proportion [20–22].

The desired body shape of amateur ballet dancers is usually idealized in the shape of professional dancers, as discussed by Leal et al. [23]. Failure to achieve such an ideal body can potentially lead to BI dissatisfaction or concern and trigger ED in amateur classical ballet dancers [18, 19]. Classical ballet practicing is commonly initiated in childhood and adolescence, with girls predominating in this modality. The practice often persists into adulthood, and most classical ballet dancers do not reach a professional level [24].

Not only ballet dancers, but women, in general, have suffered from the desire of an idealized body and overall ED prevalence may vary from 2.2 to 8.4% in women [25]. It is culturally acceptable for women to worry about their physical appearance and desire a different body. Many women exercise in gyms, seeking improvements in body shape, and gyms are places that commonly establish a culture of body worship and fast results to achieve the desired body, negatively influencing BI [26]. Nevertheless, studies have shown that practicing physical activity on a regular basis was associated with better BI and life quality [27, 28].

To the best of our knowledge, only one study has evaluated BI and the presence of behaviors for ED in female adult amateur classical ballet dancers, compared to other groups of women performing other sports non-professionally. Ravaldi et al. [29] compared modern and classical ballet dancers to other non-professional sportswomen and sedentary women. They concluded amateur performers of ballet showed more inappropriate eating attitudes and behaviors. A study compared amateur classical ballet dancers to elite professional ballet dancers, showing the latter presented higher BI distortion, dissatisfaction and ED prevalence [30]. Nonetheless, as mentioned, few classical ballet dancers reach professional dancing, and those who remain amateurs do not have practice objectives and routines comparable to professionals. Thus, the few existing studies limit knowledge on the impacts of practicing ballet non-professionally on BI and the additional risk for ED. Thus, it is essential to compare amateur ballet dancers to non-professional sportswomen.

This study aimed to evaluate if amateur female classical ballet dancers presented different BI perception and eating disordered behaviors compared to gym users and sedentary women. This comparison is essential to assess if the practice of ballet at a non-professional level is associated with behaviors for ED, and studies on this topic may lead to improved health assistance, helping to minimize the occurrence of ED. Thus, the hypothesis tested in the present study was that amateur classical ballet dancers present more BI distortion, dissatisfaction and concern; and eating disordered behaviors when compared to gym users and sedentary women.

## Methods

### Study design and ethics

This was a cross-sectional, observational and comparative study, with data collection from August 2016 to June 2018. The study received ethical approval from the Research Ethics Committee of Federal University of Rio Grande do Norte, Natal, Brazil (CAAE 38086214.2.0000.5292- # 1.753.979, Set/2016) and all participants provided written consent to participate in the study.

### Population/participants

There were four reference schools and foundations with classical ballet classes, attending dancers from child to adult ages, in all levels of classical ballet practice. Two of these schools were public and two private, and all had frequent participation in festivals and dance competitions. Eligibility criteria for the present study were amateur female adult dancers who were training in classical ballet at an intermediate/advanced level. This level of training was chosen because it could be related to more pressure in performance, possibly influencing BI and behaviors for ED [31]. Thus, training at least 6 h per week and using point shoes for at least 1 year, which characterize this level of training [32, 33], were used as eligibility criteria. It is important to mention that in this level of practice, training hours also involve time for rehearsals and artistry/theatrical training. Only adults over 19 years old were included and, in the present study, 19 non-professional ballet dancers were evaluated.

For each ballet dancer in the study, a gym user and a sedentary adult woman were scheduled for data collection. Inclusion criteria for gym users were adult women, who declared to be healthy and attended gyms at least 2 h per week for aerobic and anaerobic activities with no professional or competitive purposes. This time and type of practice was used as it possibly represented the regular practice of physical exercise in local gyms, with no competitive or professional reasons. Our population presented a mean of 4.2 h of training per week, similar to the sample described by Rossi and Tirapegui [4] in a population of female Brazilian gym users. Sedentary women were those who declared to be healthy, and that did not practice any routine physical exercises.

Thus, n = 57 women participated in this study, n = 19 of which were ballet dancers, n = 19 gym users and n = 19 sedentary (median age was of 24.0 years for the ballet dances, 25.0 years for gym users and sedentary women). None of the studied groups included pregnant women, self-reported chronic diseases, women using antidepressants, antipsychotics, anticonvulsants, or hypoglycemic drugs. There where no drop-outs in the present study.

The sample size of n = 57 was calculated assuming an overall prevalence of eating disorders in ballet dancers of 16% [14] and 2.5% in the female population [25], considering an alpha level of 0.05 and a power of 80%.

### Data collection/procedures

Screening of the population of ballet dancers in the city of Natal, Rio Grande do Norte, Brazil, was performed before recruitment. In these schools and foundations, 25 women classical ballet dancers practiced classical ballet non-professionally at intermediate/advanced levels. The eligible dancers (n = 25) were invited to participate in the study by social media, using non-probabilistic sampling. Gym users and sedentary women were recruited by social media propagation of the study, using the university social media on Facebook (around 1 thousand followers by the time of the study), using non-probabilistic sampling. Data collection occurred at the University Hospital Onofre Lopes. Participants were contacted individually, using social media tools, and were clearly informed about the study's aim. The researchers conducted the anthropometric measures, and a trained technician conducted the dual X-ray absorptiometry (DXA). Then, the researchers conducted the figure rating scale and distributed the other questionnaires, asking the participants to read and answer every item of all the questionnaires.

## Measures

### Body mass index and body composition

The body mass index (BMI) was calculated and classified as proposed by the World Health Organization [34]. Weight and height were measured using an electronic P200C Anthropometric scale (Líder®), with a capacity of 200 kg and precision of 100 g for weight, and a capacity of 2 m and accuracy of 0.1 cm for height.

Body composition was accessed by dual X-ray absorptiometry (DXA). The instrument (Lunar®, Madison WI, USA) was calibrated before the evaluations according to the manufacturer's recommendations, allowing a ± 3% coefficient of variation. Body fat percentage (%BF) and bone mineral density (BMD) at the femoral neck Z-scores were considered in the study. The examination was performed with the participant lying immobile in dorsal decubitus, with knees and ankles immobilized with a soft tape. Participants were required to remove accessories with metals and asked not to perform physical exercises or drink alcohol in the 12 h before the exams. The examination was not performed during the menstrual period of the evaluated subjects to control for body water retention. BMD at the femoral neck was classified according to the World Health Organization [35].

### Body image

Body image (BI) was assessed using the Portuguese validated version of the body shape questionnaire (BSQ) [36] and a validated Brazilian figure rating scale (FRS) [37, 38]. The BSQ is a self-administered 34-item questionnaire to measure concern with body shape and weight in the last four weeks [36]. The BSQ was developed by Cooper et al. [39], validated for the Brazilian population with good psychometric properties (α = 0.97) [36], which were also observed in the present study (α = 0.95). The results of the BSQ are classified as not concerned with BI, when < 80 points, slightly concerned when 80–110 points, moderately concerned when 110–140 points and extremely concerned when > 140 points [39].

The FRS measures body dissatisfaction and distortion. The instrument is a scale of 15 Brazilian women silhouettes, increasingly distributed according to BMI (12.5 to 47.5 kg/m^2^), showing good psychometric properties and satisfactory stability for Brazilian adults and children [37, 38]. For each numbered silhouette, there is a possible BMI, not visible to the participant. The silhouettes were randomly displayed, and the participant asked which silhouette she thought she looked like and which she desired to look. The answers were recorded, as well as the silhouette corresponding to the measured BMI.

BI distortion was assumed when the calculated BMI was not compatible with the BMI range estimated by the participant using the FRS (e.g. a participant chose the picture that represented the BMI of 27.5–30.0 to represent her current body, but her actual calculated BMI was 20.0, thus represented by another picture). BI dissatisfaction was assumed when the silhouette stated by the participant as the current body was different from the one indicated she would like to be (e.g. a participant chose the picture that represented the BMI of 27.5–30.0 to represent her current body, but she indicated she would like to be as the picture that represented the BMI of 17.5–20.0) [37, 38].

### Eating disordered behaviors

Eating disordered behaviors were assessed by the self-administered eating attitudes test (EAT-26) and the bulimic investigatory test, Edinburgh (BITE). The EAT-26 is directed at anorexic symptomatology and has been validated for the Brazilian population, with good internal consistency (α = 0.82) [40], similar to that found in the present study (α = 0.77). The EAT-26 is structured on three aspects: (1) diet (13 items), directed to the dietary restriction for foods with high caloric values, concern with avoiding food, and excessive anxiety with thinness; (2) food concerns (6 items), such as concern episodes of excessive food intake, followed by pathological methods of body weight control; (3) self-control (7 items) concerning food and the influences that the environment can have on eating habits [41]. A score < 21 points was considered a negative EAT-26 and > 21 points, positive [42].

The bulimic investigatory test, Edinburgh (BITE) was developed by Henderson and Freeman [43] to identify individuals with binge eating and assess cognitive and behavioral aspects related to bulimia, tracking and assessing the severity of bulimia. The BITE provides the symptom scale, which measures the degree of symptoms present, and the severity scale, which measures the severity of the compulsion and purgative behavior, defined by its frequency. The questionnaire showed adequate psychometric properties in the original study (α = 0.96) and in the present study (α = 0.72). The BITE was translated to Portuguese and validated for the Brazilian population [44]. A symptom score of 0–10 represented the absence of both compulsive eating and binge-eating. A symptom score of 10–19 suggested an unusual eating pattern, but not bulimia. A symptom score ≥ 20 indicated a highly disordered eating pattern and the presence of binge-eating. The severity scale ≥ 5 was considered clinically significant, and ≥ 10 indicated a high degree of severity [43].

### Statistical analysis

Continuous variables were tested for normality using the Kolmogorov–Smirnov test. Data without normal distribution were presented as median (*Mdn*) and quartiles 1 and 3 (Q1–Q3) and, with normal distribution, as mean (*M*) and standard deviations (SD). Categorical variables were presented as absolute and relative frequencies. Non-parametric variables were analyzed using the Kruskal–Wallis *H* test, followed by Dunn’s post-hoc test and the Mann–Whitney’s *U* test. The parametric variables were tested using one-way ANOVA *F* test, followed by Tukey’s post-hoc test. The Chi-square test (*X*^*2*^) was used to evaluate the frequency distributions of categorical variables. Effect sizes were computed: eta-squared (η^2^) for Kruskal–Wallis *U* test, Cohen’s d for Mann–Whitney’s *U* test and partial eta-squared (η_p_^2^) for one-way ANOVA) *F* test. Eta- (η^2^) and partial eta-squared (η_p_^2^) indicated a small effect size when 0.01, medium when 0.06 and large when 0.14. Cohen’s d indicated a small effect size when 0.20, medium when 0.50, large when 0.80 and very large when 1.30 [45].

Only fully completed questionnaires were considered in the present analysis. For the BSQ, figure rating scale and EAT-26, there were losses, and n = 56 BSQ, n = 43 figure rating scale and n = 56 EAT-26 were complete in the study. Because these could affect the power, a power analysis was then conducted a posteriori for the Chi-square test (*X*^*2*^), considering each sample size, using GPower software. The achieved power was 81% for the BSQ, 84% for the figure rating scale and 93% for the EAT-26, assuming a large effect size at 0.5 and alpha at 0.05.

Correspondence analysis was performed to explore the associations of BI and the studied groups. This approach summarizes categorical variables into a few dimensions, explaining the maximum amount of variability in the active variables included in the analysis. The objective of the analysis is to explain the largest variation (inertia), with the smallest number of dimensions, calculated by the model. Correspondence analysis is a useful qualitative tool to reveal relationships that would not be identified using other non-multivariate statistical techniques, such as performing pairwise comparisons. Assumptions are homogeneity of variance across row and column variables, no variables with zero entries, data preferably with at least three categories, and no negative values [45]. The BSQ was the only variable that met all those assumptions and thus chosen to represent the associations of BI and the studied groups. The results were represented on a map, showing each category included as a symbol, plotted in the dimensions constructed by the analysis. The closer the points, the stronger the relationship between the categories [46].

The Spearman's rho correlation (*r*_s_) was used to test the existence of a correlation between the BSQ, EAT-26 and BITE scores, and the anthropometric nutritional status (BMI) and body composition (%BF and BMD) variables.

Data analysis was performed using the *Statistical Package for Social Sciences* version 11.5 (SPSS Inc. Chicago, IL). The level of significance was set at 5%.

## Results

Ballet dancers presented significantly lower BMI, of *M* = 20.9 (*SD* = 2.4) when compared to sedentary women, of *M* = 23.2 (*SD* = 3.5) (*F*(2, 53) = 3.41, *p* = 0.040, η_p_^2^ = 0.11; Tukey’s post-hoc test, *p* = 0.031). %BF was also lower in ballet dancers when compared to sedentary women (*Mdn* = 31.2% vs. *Mdn* = 38.9%; *H*(2) = 12.78, *p* = 0.002, η^2^ = 0.67; Dunn’s post-hoc test, *p* = 0.002; Table 1). The BMD at the femoral neck was within the expected limits for age in the three studied groups, with no significant differences between them (Table 1).

**Table 1:** Characterization of the studied groups by training time, anthropometric nutritional variables and body composition

Variables	Total	Ballet dancers	Gym users	Sedentary women	*p, F (df), p,* η_p_^2^*or H (df), p,* η_p_^2^
	*Mdn* (Q1–Q3) or *M* (SD)	*Mdn* (Q1–Q3) or *M* (SD)	*Mdn* (Q1–Q3) or *M* (SD)	*Mdn* (Q1–Q3) or *M* (SD)	
Age (years)	25.0 (20.5–29.0)	24.0 (20.0–29.0)	25.0 (23.0–30.0)	25.0 (20.0–37.0)	.461
Practice time (years)	3.0 (1.0–14.3)	14.0 (6.0–17.0)	1.0 (0.5–2.0)	–	< .0005
Training time (hours/week)	5.0 (3.0–8.0)	7.5 (6.0–10.0)	3.0 (3.0–5.0)	–	< .0005
Body mass index (Kg/m^2^)	22.0 (2.9)	20.9 (2.4)*	22.1 (2.4)	23.2 (3.5)	3.41 (2, 53), .04, .11
Body fat (%)	34.4 (30.3–38.9)	31.2 (21.6–34.4)**	34.3 (30.2–38.3)	38.9 (34.2–42.2)	.002
Femoral neck BMD (Z-scores)	0.0 (0.8)	0.2 (1.2)	− 0.2 (0.6)	0.1 (0.6)	.153

Non-parametric data were analyzed using the Kruskal–Wallis *H* test, followed by the Dunn post-hoc test with effect size assessed by the eta-squared (η^2^) and the Mann Whitney *U* test with effect size assessed by Cohen’s *d*. Parametric data were analyzed by One-way ANOVA *F* test, followed by Tukey’s post-hoc test with effect size assessed by partial eta-squared η_p_^2^.

*BMD* bone mineral density

*Tukey’s HSD post-hoc test, *p* = .031. **Dunn’s post-hoc test, *p* = .002

**Fig. 1:**
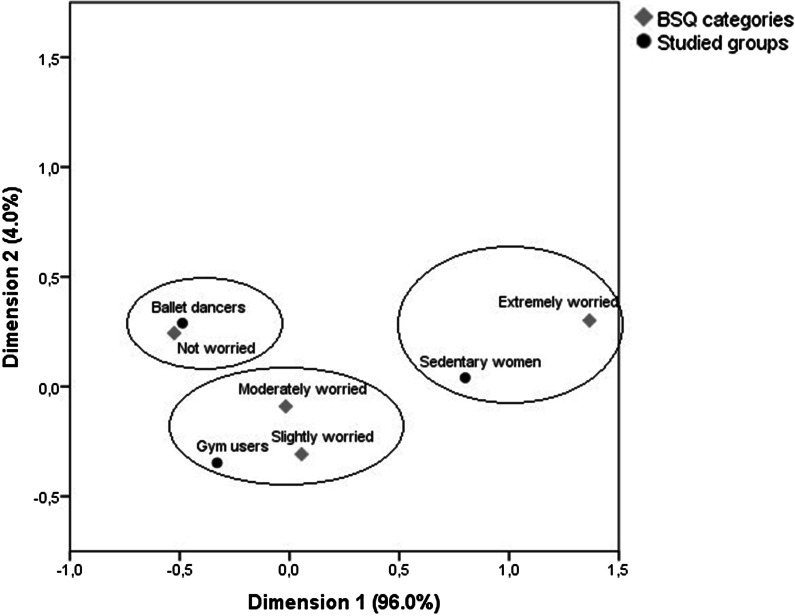
Correspondence analysis map between body image (BI) concern, assessed by the Body shape questionnaire (BSQ), and ballet dancers, gym users and sedentary women. Dots represent the classification categories of the BSQ: not concerned, slightly concerned, moderately concerned and extremely concerned with body image (BI). Lozenges represent the studied groups. The closer the symbols are, the stronger the relationship between the categories and the studied groups. Dotted circles indicate the associations found in the model. Total inertia of the model was 11.5%, and dimension 1 explained 96.0% of the model and dimension 2, 4.0%

The correspondence analysis map showed that the ballet dancers were the closest to the category of “not concerned with BI” from the BSQ. Gym users were closer to the category of “slightly concerned with BI”, while sedentary women were the closest to the category of “extremely concerned with BI”. Total inertia of the model was 11.5%, which means that belonging to one of the studied groups explains 11.5% of the concern with BI (Fig. 1).

**Fig. 2:**
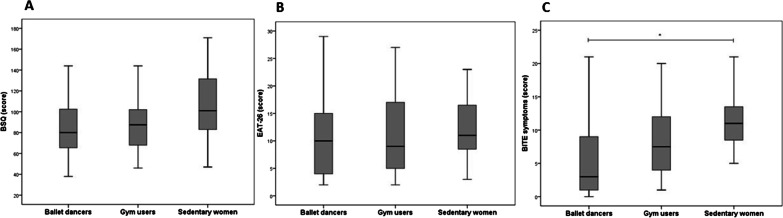
Body image and risk behaviors for eating disorders in the studied groups. **A** Body shape questionnaire (BSQ) scores—One-way ANOVA, *F(2, 53)* = 2.70, *p* = .076, η_p_^2^ = .09; **B** eating attitudes test (EAT-26) scores—Kruskal–Wallis test, *H*(2) = 1.07, *p* = .587, η^2^ = .06. **C** Bulimic investigatory test, Edinburgh (BITE) scores—One-way ANOVA, *F*(2, 54) = 5.53, *p* = .007, η_p_^2^ = .17; Tukey’s post-hoc test, *p* = .005

The overall mean BSQ score was *M* = 92.6 (SD = 30.8) points, not statistically different among the studied groups *F(2, 53)* = 2.70, *p* = 0.076, η_p_^2^ = 0.09; Fig. 2A), and indicated that the participants were slightly concerned with BI. BI distortion, evaluated by the FRS, did not differ between the groups (*X*^2^(2) = 1.56, *p* = 0.460). Most of the women thought themselves to be larger than they were, and this did not differ between the groups (*X*^2^(2) = 1.47, *p* = 0.479; Table 2). BI dissatisfaction was significantly lower (*X*^2^(2) = 6.38, *p* = 0.041; Table 2) in the ballet dancers (75.0%) and gym users (70.6%) when compared to the sedentary women (100.0%) (Table 2). Most of the women desired a smaller silhouette than they thought to have, and this did not differ between the studied groups (*X*^2^(2) = 5.22, 0.074; Table 2).

**Table 2:** Body Image and behaviors for eating disorders in the studied groups

Body Image	Total n (%)	Ballet dancers n (%)	Gym users n (%)	Sedentary women n (%)	Chi-square, *p* value
**Concern with body image (BSQ)**					
*Not concerned*	20 (35.7)	9 (47.4)	7 (38.9)	4 (21.1)	
*Slightly concerned*	20 (35.7)	6 (31.6)	7 (38.9)	7 (36.8)	.373
*Moderately concerned*	9 (16.1)	3 (15.8)	3 (16.7)	3 (15.8)	
*Extremely concerned*	7 (12.5)	1 (5.3)	1 (5.3)	5 (26.3)	
*Total*	56 (100.0)	19 (100.0)	18 (100.0)	19 (100.0)	
**Figure rating scale**					
Presence of body image distortion^1^	43 (84.3)	12 (75.0)	14 (87.5)	17 (89.5)	.460
*Perceives herself to be smaller than she is*	8 (18.6)	2 (16.7)	4 (28.6)	2 (11.8)	
*Perceives herself to be bigger than she is*	35 (81.4)	10 (83.3)	10 (71.4)	15 (88.2)	.479
*Total*	43 (100.0)	12 (100.0)	14 (100.0)	17 (100.0)	
Presence of body image dissatisfaction^2^	43 (82.7)	12 (75.0)	12 (70.6)	19 (100.0)	.041
*Wants to look smaller than she thinks she is*	31 (72.1)	11 (91.7)	6 (50.0)	14 (73.7)	
*Wants to look bigger than she thinks she is*	12 (27.9)	1 (8.3)	6 (50.0)	5 (26.3)	.074
*Total*	43 (100.0)	12 (100.0)	12 (100.0)	19 (100.0)	
**Eating disorders**					
EAT-26					
*Negative*	48 (85.7)	16 (84.2)	17 (94.4)	15 (78.9)	
*Positive*	8 (14.3)	3 (15.8)	1 (5.6)	4 (21.1)	.393
*Total*	56 (100.0)	19 (100.0)	18 (100.0)	19 (100.0)	
BITE					.044
*Symptoms scale*					
Absence of compulsive eating	33 (57.9)	15 (78.9)	12 (63.2)	6 (31.6)	
*Unusual eating pattern*	21 (36.8)	3 (15.8)	6 (31.6)	12 (63.2)	
Highly disordered eating pattern	3 (5.3)	1 (5.3)	1 (5.3)	1 (5.3)	
*Total*	57 (100.0)	19 (100.0)	19 (100.0)	19 (100.0)	
*Severity scale*					
Clinically significant	6 (100.0)	0 (0.0)	1 (100.0)	5 (100.0)	
High degree of severity	0 (0.0)	0 (0.0)	0 (0.0)	0 (0.0)	*
Total	6 (100.0)	0 (0.0)	1 (100.0)	5 (100.0)	

^1^Body image distortion was considered when the calculated BMI was not compatible with the BMI range estimated by the participant using the FRS. 2Body image dissatisfaction was considered when the silhouette stated as the current body was different from the one indicated she would like to be.

*BSQ* Body shape questionnaire, *EAT-26* eating attitudes test, *BITE* bulimic investigatory test, Edinburgh.

*Not applicable

The EAT-26 score also did not differ among the groups (*H*(2) = 1.07, *p* = 0.587, η^2^ = 0.06) with a *Mdn* = 10.0 points, indicating negative behaviors for anorexia (< 21 points). The BITE symptoms score was significantly lower in the ballet dancers when compared to the sedentary women (*M* = 5.32, *SD* = 5.6 vs. *M* = 10.9, *SD* = 4.8; *F*(2, 54) = 5.53, *p* = 0.007, η_p_^2^ = 0.17; Tukey’s post-hoc test, *p* = 0.005; Fig. 2).

In the ballet dancers, BMI and %BF were significantly and positively correlated with the BSQ score (*r*_s_ = 0.657, *p* = 0.002 and *r*_s_ = 0.574, *p* = 0.010, respectively; Table 3). In the gym users, BMD at the femoral neck was significantly and positively correlated with BMI (*r*_s_ = 0.703, *p* = 0.003; Table 3) and the BITE (*r*_*s*_ = 0.632, *p* = 0.005; Table 3); %BF was significantly and negatively correlated with the EAT-26 score (*r*_*s*_ = -0.571, *p* = 0.013; Table 3). In the sedentary women, BMI was significantly and positively correlated with the EAT-26 score (*r*_*s*_ = 0.462, *p* = 0.047; Table 3) and with the BSQ score (*r*_*s*_ = 0.653, *p* = 0.002, Table 3); and the %BF was significantly and positively correlated with the BSQ score (*r*_*s*_ = 0.653, *p* = 0.002; Table 3).

**Table 3:** Spearman’s rho correlations (*r*_s_) between nutritional variables, body image (assessed using the BSQ), and behaviors for eating disorders (assessed using EAT-26 and BITE) in the studied groups

Variables	1	2	3	4	5	6
*Ballet dancers*						
1. BMI	–					
2. %BF	0.763**	–				
3. Femoral neck BMD (Z-scores)	0.202	0.321	–			
4. BSQ	0.657**	0.574**	0.092	–		
5. EAT-26	0.426	0.280	– 0.055	0.651**	–	
6. BITE	0.194	0.140	0.329	0.090	0.193	–
*Gym users*						
1. BMI	–					
2. %BF	0.009	–				
3. Femoral neck BMD (Z-scores)	0.703**	0.027	–			
4. BSQ	0.351	0.013	0.488	–		
5. EAT-26	– 0.100	– 0.571*	0.179	0.538*	–	
6. BITE	0.234	0.009	0.689**	0.501*	0.342	–
*Sedentary women*						
1. BMI	–					
2. %BF	0.719**	–				
3. Femoral neck BMD (Z-scores)	0.108	0.180	–			
4. BSQ	0.653**	0.524*	– 0.145	–		
5. EAT-26	0.462*	0.421	– 0.299	0.673**	–	
6. BITE	0.355	0.229	0.001	0.543	– 0.114	–

*BSQ* body shape questionnaire, *EAT-26* eating attitudes test, *BITE* bulimic investigatory test, Edinburgh; *BMI* body mass index, *%BF* body fat percentage, *BMD* bone mineral density.

**p < .01; *p < .05

## Discussion

Our results showed that ballet dancers presented less BI dissatisfaction compared to the sedentary women and lower behaviors for bulimia compared to the other studied groups. There were no differences in BI distortion and behaviors for anorexia between the studied groups. To the best of our knowledge, this is the first study to compare these variables in non-professional women ballet dancers with gym users and sedentary women.

Ballet dancers showed the lowest BMI and %BF, in agreement with other studies [47, 48]. Classical ballet requires hours of rehearsal, even to non-professionals [49], as demonstrated by our results, increasing the energy demand and affecting nutritional status [47]. Low BMD has been associated with disordered eating behaviors and BI in ballet dancers, even in non-professionals [50–52]. In the present study, BMD was within the expected limits for age in the ballet dancers, demonstrating no negative impact of ballet practicing on BMD in the studied population.

Similar BI distortion was observed in the studied groups and BI dissatisfaction was significantly lower in the ballet dancers compared to the sedentary women. This result might be explained by the fact that the studied ballet dancers practiced classical ballet for many years, many hours/week. This practice was associated with a thinner body, as demonstrated by our results. This lean body puts the studied ballet dancers closer to the shape idealized by most of the women [53, 54].

The correspondence analysis reinforced that having a leaner body is associated with better BI because ballet dancers were closer to the “not concerned with BI” category. Sedentary women, in turn, were more distant from the body considered ideal and, therefore, more dissatisfied, closer to the “extremely concerned with BI” category. Other factors might explain BI concern in women, and these vary among the studied populations. Keirns and Hawkins [55] have found that intuitive eating (a positive psychosocial pattern of adaptive eating) and BI concern are moderated by BMI, with higher BMI values decreasing this relationship. These results reinforce that presenting a leaner body decreased BI concern in the studied women.

Fewer bulimic behaviors in ballet dancers and no difference in behaviors for anorexia compared to the other studied groups were observed in the present study. The behaviors for ED were not exclusive to the ballet dancers, and we also found high percentages of BI dissatisfaction and distortion in gym users and sedentary women. Thus, our results refuted the initial hypothesis that BI distortion, dissatisfaction and concern; and behaviors for ED would be more present in the ballet dancers. These behaviors were more present in sedentary women.

Some studies evaluated BI and eating disordered behaviors in ballet dancers, compared to other groups, and found different results [56, 57]. A study compared adolescent ballet dancers with basketball players and non-athletes and found impaired physical self-concept and disturbed eating behaviors in ballet dancers and basketball players [56]. A study with adult models, ballet dancers and young students found the adult models and ballet dancers presented higher BI distortion, higher scores for the ED inventory and neurotic perfectionism symptoms [57].

We studied adult amateur ballet dancers, who may not be highly pressed to have a perfect performance [21]. Thus, practicing ballet in an amateur long term environment might be beneficial to BI. On the other hand, perhaps sedentary women expect themselves to have an ideal body but without adopting a healthier lifestyle. Although there is vast literature regarding the prevalence of overall eating disorders in the female population [25] unusual eating patterns and body image have not been extensively studied in sedentary adult female, most of the studies focus on adolescents and/or athletes [9, 12, 18, 26, 58–60].

A cross-sectional study among adults aged 34–65 years (n = 13,286) found almost 90% of body dissatisfaction in women with low physical activity. Those with high physical activity presented less body dissatisfaction (almost 80%) [27]. Thus, being sedentary may negatively affect mental health, and practicing a long term, regular exercise such as ballet dancing in an amateur setting may be actually beneficial to mental and social health.

The profile of dance schools may also contribute to the vulnerability of ballet dancers to ED. There are non-professional dance schools where a lean BI is not promoted. Learning to dance in this context can positively influence BI because even non-professional ballet dancers tend to have a leaner body, as demonstrated by our results, and it positively affects BI [61]. In this study, although the ballet dancers were from different schools and foundations, these were all non-professional.

BMI and %BF were positively correlated with BI concern in the ballet dancers and sedentary women. Some studies suggest that the higher the BMI and %BF, the greater BI dissatisfaction, confirming that women aim for a slim body [62, 63]. %BF was negatively correlated with behaviors for anorexia in gym users. This result is also consistent with the literature showing that a lower %BF may be related to a higher chance of developing ED [18].

BMI and BSQ were positively correlated with the BMD at the femoral neck in gym users. The direct relationship between bone metabolism and body weight is already well recognized in the literature; low body weight is associated with lower bone mass, just as overweight is associated with higher bone mass [64–66]. In the study by Rassy et al., young women with low weight had lower bone mineral density at the femoral neck than women with adequate and high BMI [64]. In the present study, we found a positive correlation between BITE and BMD at the femoral neck in gym users, and further studies are needed to clarify this relationship.

Some limitations of the present study should be mentioned, such as the small sample size and non-probabilistic sampling. Because intermediate and advanced levels of ballet practice turn to be scarce, female amateur ballet dancers were screened and non-probabilistic sampling used to recruit for the present research. Nevertheless, these groups of female dancers are quite homogeneous, as discussed by Arcelus et al. [16]. The number of epidemiological studies in the field of dance seeking to understand BI and eating disordered behaviors are limited and might be explained by the complexity of the groups of ballet dancers, which turn to be scarce in the intermediate and advanced levels of practice [16, 57]. Thus, most studies, instead of determining the prevalence of ED, associate ED with other factors, such as menstruation or injuries, without adequate comparison with other groups. In addition, athletes at different levels tend to underestimate ED symptoms when using self-report measures [67]. These limitations impair understanding the occurrence of ED in dancers, especially those who are adults and non-professionals, as is the case in the present study.

The fact that the gym users and sedentary women volunteers were recruited by the university social media might have given selection bias once young women in social media might be more concerned about BI. A strength of this study is the comparison of the ballet dancers with gyms users, which could also present a higher risk for ED and sedentary women, who did not practice physical activities related to ED. Although dancers are recognized as a risk group for ED, BI distortion/dissatisfaction and the behaviors for ED were not more present in the studied ballet dancers. Data presented clinical relevance because there is a tendency to automatically imagine more risk for ED in ballet dancers, regardless of the training context.

## Conclusions

Data suggested that amateur classical ballet practicing is associated to better BI and fewer behaviors for ED in the studied population. The lower BMI in ballet dancers might explain these findings, and further studies should explore these associations more.

## Data Availability

The data presented in this study are available on request from the corresponding author.

## Abbreviations

%BF: Body fat percentage
BI: Body image
BITE: Bulimic investigatory test, Edinburgh
BMD: Bone mineral density
BMI: Body mass index
BSQ: Body shape questionnaire
DXA: Dual X-ray absorptiometry
EAT-26: Eating attitudes test
ED: Eating disorders
FRS: Brazilian figure rating scale
